# *CD40:* Novel Association with Crohn's Disease and Replication in Multiple Sclerosis Susceptibility

**DOI:** 10.1371/journal.pone.0011520

**Published:** 2010-07-12

**Authors:** Fiona Blanco-Kelly, Fuencisla Matesanz, Antonio Alcina, María Teruel, Lina M. Díaz-Gallo, María Gómez-García, Miguel A. López-Nevot, Luis Rodrigo, Antonio Nieto, Carlos Cardeña, Guillermo Alcain, Manuel Díaz-Rubio, Emilio G. de la Concha, Oscar Fernandez, Rafael Arroyo, Javier Martín, Elena Urcelay

**Affiliations:** 1 Department of Clinical Immunology, Hospital Clínico San Carlos, Madrid, Spain; 2 Instituto Parasitología y Biomedicina “López Neyra”, C. S. I. C., Granada, Spain; 3 Servicio de Digestivo, Hospital Universitario Virgen de las Nieves, Granada, Spain; 4 Servicio de Inmunología, Hospital Universitario Virgen de las Nieves, Granada, Spain; 5 Servicio de Digestivo, Hospital Universitario Central de Asturias, Oviedo, Spain; 6 Servicio de Inmunología, Hospital Puerta del Mar, Cádiz, Spain; 7 Servicio de Digestivo, Hospital Clínico San Cecilio, Granada, Spain; 8 Servicio de Digestivo, Hospital Virgen de la Victoria, Málaga, Spain; 9 Digestive Department, Hospital Clínico San Carlos, Madrid, Spain; 10 Servicio de Neurología, Instituto de Neurociencias Clínicas, Hospital Carlos Haya, Málaga, Spain; 11 Multiple Sclerosis Unit, Neurology Department, Hospital Clínico San Carlos, Madrid, Spain; 12 Members of the Red Española de Esclerosis Múltiple (REEM), www.reem.es; Instituto de Biomedicina de Valencia, CSIC, Spain

## Abstract

**Background:**

A functional polymorphism located at −1 from the start codon of the *CD40* gene, rs1883832, was previously reported to disrupt a Kozak sequence essential for translation. It has been consistently associated with Graves' disease risk in populations of different ethnicity and genetic proxies of this variant evaluated in genome-wide association studies have shown evidence of an effect in rheumatoid arthritis and multiple sclerosis (MS) susceptibility. However, the protective allele associated with Graves' disease or rheumatoid arthritis has shown a risk role in MS, an effect that we aimed to replicate in the present work. We hypothesized that this functional polymorphism might also show an association with other complex autoimmune condition such as inflammatory bowel disease, given the CD40 overexpression previously observed in Crohn's disease (CD) lesions.

**Methodology:**

Genotyping of rs1883832C>T was performed in 1564 MS, 1102 CD and 969 ulcerative colitis (UC) Spanish patients and in 2948 ethnically matched controls by TaqMan chemistry.

**Principal Findings:**

The observed effect of the minor allele rs1883832T was replicated in our independent Spanish MS cohort [p = 0.025; OR (95% CI) = 1.12 (1.01–1.23)]. The frequency of the minor allele was also significantly higher in CD patients than in controls [p = 0.002; OR (95% CI) = 1.19 (1.06–1.33)]. This increased predisposition was not detected in UC patients [p = 0.5; OR (95% CI) = 1.04 (0.93–1.17)].

**Conclusion:**

The impact of *CD40* rs1883832 on MS and CD risk points to a common signaling shared by these autoimmune conditions.

## Introduction

In the past three years genome-wide association studies have identified literally hundreds of genetic loci involved in the susceptibility conferred to complex inherited traits. Even though this scenario represents an extraordinary advance in complex disease genetics, the modest effect sizes of the common polymorphisms found associated explain only a small fraction of the heritability in most of these multifactorial conditions, suggesting that many more loci remain to be discovered. One of the genes encoding a member of the tumor necrosis factor receptor family that plays a key role in adaptive immunity is *CD40* (MIM*109535) [1]. T-cell priming and B-cell activation can occur in the absence of the CD40/CD40-ligand costimulatory signal, but many immune functions are impaired without this interaction, underscoring its importance for an adequate immune response.

Candidate gene studies reported the association of a functional *CD40* polymorphism with Graves' disease; moreover, the association of this variant was replicated in populations of different ethnicity including Caucasians, Koreans, and Japanese [2]–[4]. This functional polymorphism, rs1883832, is located at −1 from the ATG within a Kozak sequence, a stretch of nucleotides essential for translation that is flanking the start codon in vertebrate genes [5]. The common allele of rs1883832 increased the translational efficiency of CD40 transcripts, resulting in 15–32% more CD40 protein than in the presence of the minor allele [6]. *CD40* has been recently associated with rheumatoid arthritis through the meta-analysis of two genome-wide studies conducted in European populations [7]. The common allele frequency of a polymorphism located in the second intron of the *CD40* gene, rs4810485 and which was in strong linkage disequilibrium with rs1883832 (r^2^ = 0.95), was reduced in arthritic patients when compared to healthy controls. The broad functionality of CD40 on immune responses, coupled with its critical role in several experimental autoimmune conditions, such as collagen induced arthritis [8], experimental Graves' disease [9], experimental autoimmune encephalomyelitis (EAE), a model of multiple sclerosis (MS) [10], lupus nephritis [11] and type 1 diabetes [12] suggest its association with other immune-mediated diseases. However, no association has been detected with systemic lupus erythematosus [13] or with type 1 diabetes [14] and, therefore, it would be interesting to ascertain the diseases associated with the *CD40* gene.

A recently performed genome-wide association study in MS identified two genetically equivalent polymorphisms (r^2^ = 1) in the 5′ region of the *CD40* gene in strong linkage disequilibrium with rs1883832C>T (r^2^ = 0.95) [15]. Interestingly, the susceptibility allele described for Graves' disease or rheumatoid arthritis seemed to protect against MS, suggestive of a different molecular mechanism involved in the aetiology of these conditions. Our aim with the present study was to replicate this association with MS and to confirm the protective effect of the common C allele at position −1 of the *CD40* gene.

Additionally, we pursued to test the effect of this polymorphism in inflammatory bowel disease. Antibodies blocking CD40 have been reported to dampen the severity of experimental colitis [16] and a CD40 overexpression in Crohn's disease lesions has been known for a decade [17]. Thus, we decided to focus attention on this component of the CD40/CD40-ligand costimulatory pathway and to investigate the association of the functional polymorphism of the *CD40* gene in the two main clinical phenotypes of inflammatory bowel disease, Crohn's disease (CD) and ulcerative colitis (UC).

## Materials and Methods

Spanish patients (1564 MS, 1102 CD and 969 UC) and 2948 ethnically matched controls, mostly blood donors and staff, were consecutively recruited from the following hospitals: H. Clínico S. Carlos (Madrid), H. Virgen Macarena (Sevilla), H. Carlos Haya and H. Virgen de la Victoria (Málaga), H. Clínico S. Cecilio and H. Virgen de las Nieves (Granada), H. Puerta del Mar (Cádiz) and the Blood Bank (Granada). MS patients were diagnosed based on the Poser criteria [18] and 37% of patients carried the HLA DRB1*1501 allele. Most patients were relapsing remitting (79%), 19% secondary progressive and 2% primary progressive. Their mean age at MS onset was 30±10 years. Diagnosis of IBD patients was based on standard clinical, radiologic, endoscopic and histologic criteria [19]. Some UC patients suffered from pancolitis (29%), extraintestinal manifestations (44%) or colectomy (27%). CD patients were classified according to the location of the lesions in ileal (L1, 40%), colonic (L2, 19%), ileocolonic (L3, 36%) and upper gastrointestinal tract (L4, 4%) and according to the disease behaviour in inflammatory (B1, 41%), stricturing (B2, 19%) and perforating (B3, 40%). Subjects were included in the study after informed consent. The Ethics Committee of the participant hospitals approved the study.

Genotyping of the samples was carried out with a pre-designed TaqMan Assay from Applied Biosystems in a 7900HT Fast Real-Time PCR system, under the conditions recommended by the manufacturer (Applied Biosystems, Foster City, CA, USA). Genotyping call-rate success was over 96% for all patient groups and controls.

The statistical analysis to compare allelic and genotypic distributions was performed using chi-square test or Fisher's exact test included in a standard statistical package (Epi Info v. 5; World Health Organization, Geneva, Switzerland) which was also used for statistical power calculations. Odds ratios (OR) and their 95% confidence intervals were estimated using the Cornfield method. Linkage disequilibrium was measured by r^2^.

## Results

Table 1 summarizes genotypic and allelic frequencies of the functional *CD40* polymorphism rs1883832 in Spanish patients suffering from multiple sclerosis, Crohn's disease or ulcerative colitis and in ethnically-matched healthy controls. Results conformed to Hardy-Weinberg expectations. As shown, the minor allele frequency and the number of carriers of this minor allele were higher in both MS and CD patients than in controls. This increased susceptibility was not observed in UC patients. Being this result in MS a replication of the original finding, there is no need for correction. The significant result obtained in CD withstands Bonferroni's correction (x4).

**Table 1:** Genotypic and allelic frequencies of the C/T_−1_ polymorphism of the *CD40* gene (p and OR values are shown for T allele and carriers of T allele).

	MS		CD		UC		Controls		MS vs. controls		CD vs. controls		UC vs. controls	
rs1883832	n	%	n	%	n	%	n	%	p value	OR (95% CI)	p value	OR (95% CI)	p value	OR (95% CI)
**TT**	13	9	107	10	66	7	237	8						
**TC**	7	41	452	42	398	42	1098	38	0.018	1.16 (1.02−1.32)	0.002	1.24 (1.08−1.43)	0.13	1.12 (0.96−1.30)
**CC**	62	50	527	48	485	51	1562	54						
**T**	89	29	666	31	530	27	1572	27	0.025	1.12 (1.01−1.23)	0.002	1.19 (1.06−1.33)	0.5	1.04 (0.93−1.17)
**C**	9	71	1506	69	1368	73	4222	73						

No difference was observed in MS, CD and UC patients stratified by the well-known susceptibility factors HLA-DRB1*1501, NOD2/*CARD15* or HLA-DRB1*103, respectively (data not shown).

## Discussion

The CD40/CD40-ligand pathway is a key component of the pathophysiology of numerous autoimmune disorders [20]. Its contribution to autoimmunity could involve different processes. The physiological relevance of CD40/CD40-ligand in Th17-cell differentiation has been recently proven using a mouse model of experimental autoimmune encephalomyelitis and *Cd40^−/−^* mice were found to be protected from the disease [21]. Increased expression of the main cytokine produced by these T cells, IL-17, has been shown in immune and inflammatory diseases including rheumatoid arthritis, multiple sclerosis and inflammatory bowel diseases [22]–[25]. Moreover, the functional blockade of CD40 with a murine antibody effectively prevents clinical expression in an animal model of multiple sclerosis [26]. Indeed, the preclinical evaluation of a monoclonal antibody against CD40 has shown beneficial activities in an MS model when administered early in the disease development, as well as after the onset of brain inflammation [27]. As mentioned, two of the three most associated polymorphisms identified in a recent genome-wide association study in MS [15] are proxies of the functional polymorphism at −1 of the *CD40* gene. However, the minor allele of this variant that decreases the efficiency of CD40 translation led to an increased susceptibility towards the disease and we decided to replicate this effect of the *CD40* polymorphism in an independent MS cohort. Our present data validate the original finding and corroborate the association of the minor allele of this polymorphism in MS risk. Indeed, Buck *et al.* [28] observed a similar increase in minor allele frequency in patients when compared to controls (33% vs. 29%), although a limited statistical power prevented them from reaching the significance threshold.

Then, we aimed at addressing the influence of the functional polymorphism at −1 of the *CD40* gene in inflammatory bowel disease risk. Our data evidenced a parallel pattern of association of CD and MS, with the minor allele conferring susceptibility to both conditions. No significant effect of this polymorphism was detected in the other main subphenotype of inflammatory bowel diseases, UC, although the size of our collection would allow to detect the effect previously described for the minor allele in MS risk (OR = 1.18) with an 80% power (α = 0.05). A pattern of CD susceptibility similar to the one herein described for the polymorphism at −1 in *CD40* was evidenced in the genome-wide study performed in CD by the Wellcome Trust Case Control Consortium [29], where rs4810485 (proxy of rs1883832, r^2^ = 0.95) was associated with CD [p = 0.009; OR (95%CI) = 1.14 (1.03–1.25)]. However, the significance threshold imposed in this type of whole-genome scans hampered the consideration of this signal as a CD susceptibility factor. Interestingly, in this WTCCC study, the minor allele revealed a borderline significant signal of opposite effect in rheumatoid arthritis [p = 0.067; OR (95%CI) = 0.91 (0.83–1.01)], in agreement with the meta-analysis recently published (OR = 0.87, [7]). Therefore, the present data support the consideration of CD40 as a genuine risk factor for CD. Moreover, a significant difference between the allelic frequencies of this polymorphism in CD and UC patients is observed (p = 0.027), concordantly with a recent study showing that only half of the known genes associated with CD are shared by UC [30].

As recently published by Zheng J et al. [31], CD8(+) Treg play important roles in the maintenance of immune tolerance. Adoptive transfer of these CD8(+) Treg in rodents or induction of CD8(+) Treg in humans can prevent or treat autoimmune diseases. CD8(+) T cell subsets could be induced from naive CD8(+) precursors in vitro by allogenic CD40-activated B cells. Moreover, the Tregs induced by CD40-B have greater suppressive capacity and are generated in larger numbers than those induced by immature dendritic cells [32]. Provided that the minor allele of the studied *CD40* variant decreases the efficiency of translation, this lower amount of CD40 would then result in a reduced induction of Treg, which would lead to a disruption of immune tolerance. Further research regarding the balance of effector and regulatory T cells will help to ascertain the pleiotropic action of CD40 signalling exerted on several immune-mediated diseases, and provide clues for the successful translation of new therapeutics.
