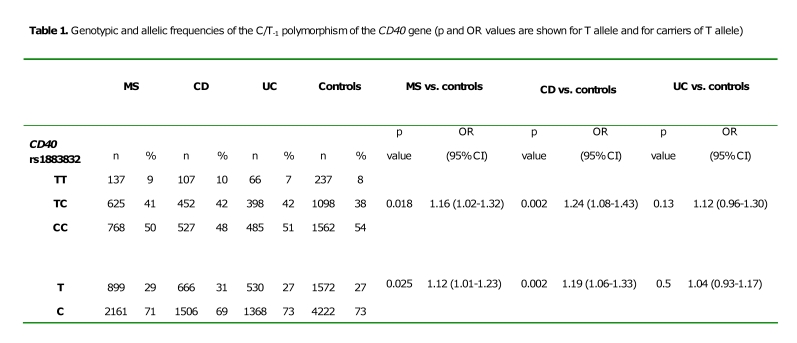# Correction: *CD40:* Novel Association with Crohn's Disease and Replication in Multiple Sclerosis Susceptibility

**DOI:** 10.1371/annotation/4d830d1b-46ff-4d72-b577-6a3765d335fc

**Published:** 2010-08-18

**Authors:** Fiona Blanco-Kelly, Fuencisla Matesanz, Antonio Alcina, María Teruel, Lina M. Díaz-Gallo, María Gómez-García, Miguel A. López-Nevot, Luis Rodrigo, Antonio Nieto, Carlos Cardeña, Guillermo Alcain, Manuel Díaz-Rubio, Emilio G. de la Concha, Oscar Fernandez, Rafael Arroyo, Javier Martín, Elena Urcelay

The values in the first column of table 1 were published incorrectly. Please view the corrected table here: 

**Figure:**